# Protective Effects of Carvedilol and Vitamin C against Azithromycin-Induced Cardiotoxicity in Rats via Decreasing ROS, IL1-*β*, and TNF-*α* Production and Inhibiting NF-*κ*B and Caspase-3 Expression

**DOI:** 10.1155/2016/1874762

**Published:** 2016-05-05

**Authors:** Nagla A. El-Shitany, Karema El-Desoky

**Affiliations:** ^1^Department of Pharmacology and Toxicology, Faculty of Pharmacy, King Abdulaziz University, Elkandarh, Jeddah 21589, Saudi Arabia; ^2^Department of Pharmacology and Toxicology, Faculty of Pharmacy, Tanta University, Tanta, Egypt; ^3^Department of Pathology, Faculty of Medicine, Tanta University, Tanta, Egypt

## Abstract

The Food and Drug Administration recently warned of the fatal cardiovascular risks of azithromycin in humans. In addition, a recently published study documented azithromycin-induced cardiotoxicity in rats. This study aimed to justify the exact cardiovascular events accompanying azithromycin administration in rats, focusing on electrocardiographic, biochemical, and histopathological changes. In addition, the underlying mechanisms were studied regarding reactive oxygen species production, cytokine release, and apoptotic cell-death. Finally, the supposed protective effects of both carvedilol and vitamin C were assessed. Four groups of rats were used: (1) control, (2) azithromycin, (3) azithromycin + carvedilol, and (4) azithromycin + vitamin C. Azithromycin resulted in marked atrophy of cardiac muscle fibers and electrocardiographic segment alteration. It increased the heart rate, lactate dehydrogenase, creatine phosphokinase, malondialdehyde, nitric oxide, interleukin-1 beta (IL1-*β*), tumor necrosis factor alpha (TNF-*α*), nuclear factor kappa beta (NF-*κ*B), and caspase-3. It decreased reduced glutathione, glutathione peroxidase, and superoxide dismutase. Carvedilol and vitamin C prevented most of the azithromycin-induced electrocardiographic and histopathological changes. Carvedilol and vitamin C decreased lactate dehydrogenase, malondialdehyde, IL1-*β*, TNF-*α*, NF-*κ*B, and caspase-3. Both agents increased glutathione peroxidase. This study shows that both carvedilol and vitamin C protect against azithromycin-induced cardiotoxicity through antioxidant, immunomodulatory, and antiapoptotic mechanisms.

## 1. Introduction

Azithromycin is a widely and effectively used macrolide antibiotic. It is used in the treatment of various types of serious bacterial infections. Recently, the Food and Drug Administration (FDA) Adverse Event Reporting System included at least 20 reports of torsades de pointes and sudden death cases associated with azithromycin 5-day treatment courses [1, 2]. On May 17, 2012, the FDA warned of the fatal cardiovascular risks of azithromycin [1]. The FDA called patients to consult their doctors about the use of azithromycin but not to stop using the drug. Additional warnings were further added to azithromycin product labels [3]. Azithromycin caused changes in electrocardiographic (ECG) parameters, particularly QT-interval prolongation. In addition, some patients developed the malignant arrhythmia known as torsade de pointes [3–11]. For the safe use of azithromycin, the FDA recommended that a baseline ECG should be conducted to establish any QT-interval abnormality. In addition, the FDA warned of the use of azithromycin with other drugs which could induce QT-interval prolongation [12–15].

A recently published study documented azithromycin-induced cardiotoxicity in rats [16]. The study reported that azithromycin-induced cardiotoxicity was accompanied by increased reactive oxygen species (ROS) generation and QT-interval prolongation [16].

This gives rise to the hypothesis that azithromycin-induced fatalities may result from QT-interval prolongation and ROS generation. We assume that if we prevent QT-interval prolongation and/or ROS generation, we will decrease the risk of life-threatening arrhythmia induced by azithromycin administration and hence decrease the mortality rate.

Carvedilol is a nonselective *β*-blocker that also possesses antioxidant effects. It improves myocardial function, increases survival, and decreases mortality in adults with congestive heart failure [17, 18]. A recent study showed that carvedilol decreases QTc-minimum, QTc-maximum, and QTd values, improves heart rate variability and reduces arrhythmia in children with idiopathic dilated cardiomyopathy [19].

Vitamin C is a potent ROS scavenger [20]. It can also regenerate other small molecule antioxidants, such as GSH [21]. Vitamin C significantly lowers exercise-induced QT-interval dispersion (QTd) after myocardial infarction [22].

This study aims to justify the exact cardiovascular events accompanying azithromycin administration in rats by focusing on electrocardiographic (ECG), biochemical, and histopathological changes. In addition, the underlying mechanisms will be studied regarding ROS production, cytokine release, and apoptotic cell-death. Finally, the probable protective effects of both carvedilol and vitamin C will be assessed.

## 2. Materials and Methods

### 2.1. Chemicals

Zithromax (250 mg azithromycin, Pfizer Pharma GmbH, Germany), Dilatrend (25 mg carvedilol, Roche, Italy), vitamin C, and urethane (Sigma-Aldrich Inc., USA) were used in this study.

### 2.2. Animals and Treatments

Adult male Sprague-Dawley rats (180–200 g) were caged at 25 ± 3°C and a relative humidity of ~50%. They were fed with standard laboratory chow and water* ad libitum*. The animals received care according to institutional guidelines for the care and use of laboratory animals at King Fahd Medical Research Center. We also followed the instructions of the Saudi National Committee of Ethics concerning animal research. The Committee of Biomedical Ethics of Research, Faculty of Medicine, King Abdulaziz University, approved the experimental design (Permit number 153-15).

### 2.3. Experimental Protocol

The rats were randomly divided into 4 groups (*n* = 6): (1) control: normal saline, per oral (po) administration, (2) azithromycin: azithromycin (10 mg/kg, po), (3) carvedilol + azithromycin: carvedilol (10 mg/kg, po) [23] + azithromycin (10 mg/kg, po), and (4) vitamin C + azithromycin: vitamin C (20 mg/kg, po) [24] + azithromycin (10 mg/kg, po). The rats were pretreated with normal saline (0.9%), carvedilol, and vitamin C for 5 days before azithromycin administration and 5 days thereafter. At the end of the experiment, the rats fasted for 12 hours and then were anesthetized with an intraperitoneal (ip) injection of urethane (1.5 g/kg) [25].

### 2.4. Electrocardiography (ECG) Assessment

The standard limb lead II of the surface ECG was recorded for each rat by the PowerLab system (ADI Instruments) connected to a PC running LabChart professional software (version 7.3) containing an ECG module. The ECG record measures the following parameters: heart rate in beats per minute (BPM), P duration, RR, QRS, QTc, Tpeak-Tend intervals in seconds (s), ST height, and T, Q, and S amplitude millivolts (mv) [26].

### 2.5. Sample Collection

On day 6 after the ECG recording, heart samples were collected and kept either frozen (−80°C) or in buffered formalin solution (10%). In addition, blood samples were withdrawn for plasma separation and storage at −80°C.

### 2.6. Measurement of Plasma Lactate Dehydrogenase (LDH) and Creatine Phosphokinase (CPK)

Total plasma LDH and CPK activities were measured using kits of Biodiagnostic, Egypt. LDH activity was measured according to Pesce [27]. The method depends on the reaction of lactate with NAD, and the NADH formed was measured spectrophotometrically at 340 nm. The increase in absorbance was measured at 1-minute intervals for 3 minutes. Plasma total LDH activity was calculated as units per liter (U/L). Total CPK activity was determined according to Abbot et al. [28]. The method depends on the transphosphorylation of ADP to ATP through a series of coupled enzymatic reactions; the NADH produced was directly proportional to the CPK activity. The increase in absorbance at 1-minute intervals was recorded for 3 minutes at 340 nm. Plasma total CPK activity was calculated as U/L.

### 2.7. Measurement of Myocardial Muscle Lipid Peroxide (Measured as Malondialdehyde (MDA)), Reduced Glutathione (GSH), and Nitric Oxide (NO)

Myocardial muscle MDA, GSH, and NO contents were measured using Biodiagnostic kits (Egypt). MDA (nmol/g tissue) was measured according to the method of Uchiyama and Mihara [29]. Briefly, thiobarbituric acid was added to the tissue homogenate and boiled in a water bath and then the color that was formed was extracted with n-butanol and measured at 535 nm and 525 nm.

The level of GSH (*μ*mol/g tissue) was determined according to the method described by Ellman [30]. This assay was based on the reduction of bis-(3-carboxy-4-nitrophenyl) disulfide reagent by the thiol group to form 2-nitro-5-mercaptobenzoic acid. The absorbance of the yellow color formed was measured spectrophotometrically at 412 nm.

NO (*μ*mol/g tissue) was determined according to the method described by Tarpey et al. [31]. NO was assayed spectrophotometrically (550 nm) by measuring total nitrate plus nitrite (NO_3_^−^ + NO_2_^−^) using Griess reagent.

### 2.8. Measurement of Myocardial Muscle Enzyme Activity of Glutathione Peroxidase (GPx), Superoxide Dismutase (SOD), and Catalase (CAT)

All the activities were determined using Biodiagnostic kits (Egypt). Myocardial muscle GPx activity was determined according to Paglia and Valentine [32]. GPx activity was determined by measuring the rate of NADPH oxidation at 340 nm using H_2_O_2_ as the substrate. GPx activity was expressed in U/g tissue.

Myocardial muscle SOD activity was determined according to Nishikimi et al. [33]. This assay depended on the ability of the SOD to inhibit the phenazine methosulphate-mediated reduction of nitro-blue tetrazolium dye. SOD activity was expressed in U/g tissue.

Myocardial muscle CAT activity was quantified according to Aebi [34]. The test was based on the reaction of H_2_O_2_ with 3,5-dichloro-2-hydroxybenzene sulfonic acid and 4-aminophenazone, producing a colored chromophore that was measured at 510 nm. CAT activity was expressed in U/g tissue.

### 2.9. Measurement of Plasma Interleukin-1 Beta (IL-1*β*) and Tumor Necrosis Factor Alpha (TNF-*α*)

TNF-*α* and IL-1*β* concentrations were measured using ELISA assay kits (Assaypro, USA). Cytokine concentrations were calculated using standard purified recombinant cytokines.

### 2.10. Histopathological Examination of the Heart

Heart sections embedded in paraffin wax were serially sectioned (3–5 *μ*m) and were H&E stained. Stained heart sections (10 high-power fields per section) of all groups were examined to assess the myocardial muscle.

### 2.11. Immunohistochemical Determination of Caspase-3 and Nuclear Factor-Kappa B (NF-*κ*B) in the Heart Sections

Immunohistochemical staining was done using kits obtained from Lab Vision (Fremont, CA). An immunoperoxidase (PAP, peroxidase/antiperoxidase) technique was adopted. The cytoplasm of each caspase-3 and NF-*κ*B (+) cell was stained brown. The brown staining was graded as follows: no brown color (−) (negative), faint brown staining (±) (mild positivity), moderate brown staining (++) (moderate positivity), and strong brown staining (+++) (marked positivity).

### 2.12. Statistical Analysis

Statistical analyses of data were carried out using Minitab Inc. software (13.1). Data were expressed as the mean ± SDM. Comparisons between groups were made with one-way analysis of variance (ANOVA) followed by the Tukey-Kramer test. Statistical significance occurred at *p* ≤ 0.05.

## 3. Results

### 3.1. Effects of Carvedilol and Vitamin C on Azithromycin-Induced Changes in the Electrocardiographic (ECG) Parameters and Patterns Measured in Azithromycin-Treated Rats

**Figure 1:**
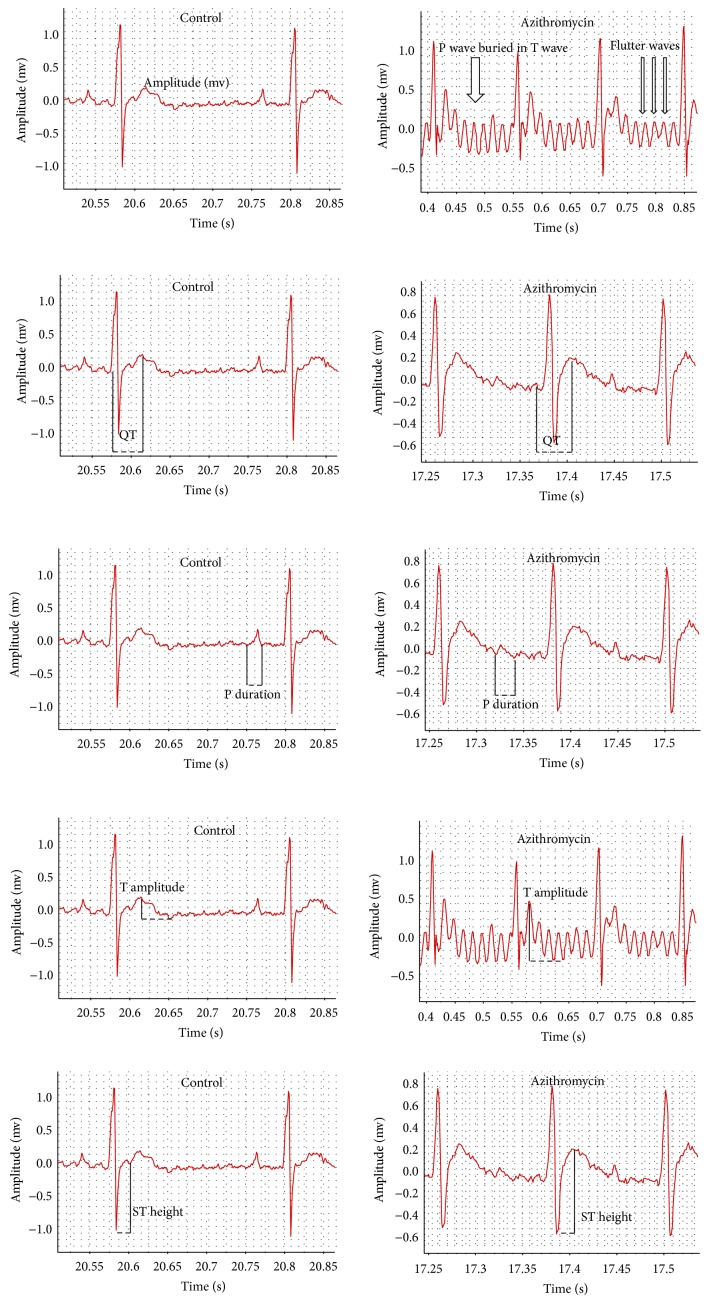
Effect of azithromycin on the electrocardiographic (ECG) pattern measured in rats.

The control rats showed a normal-pattern ECG, whereas rats treated with azithromycin (10 mg/kg) showed P waves buried in T waves, flutter waves, prolonged QT and P wave durations, and increased T amplitude and ST height (Figure 1). Treatments of rats with azithromycin significantly increased the heart rate (*p* = 0.000), P duration (*p* = 0.017), QT interval (*p* = 0.001), QTc (*p* = 0.001), Tpeak-Tend interval (*p* = 0.016), ST height (*p* = 0.029), and T amplitude (*p* = 0.049) compared to the control values (Table 1). On the other hand, treatments of rats with azithromycin significantly decreased the RR interval (*p* = 0.016) compared to the control value (Table 1).

**Figure 2:**
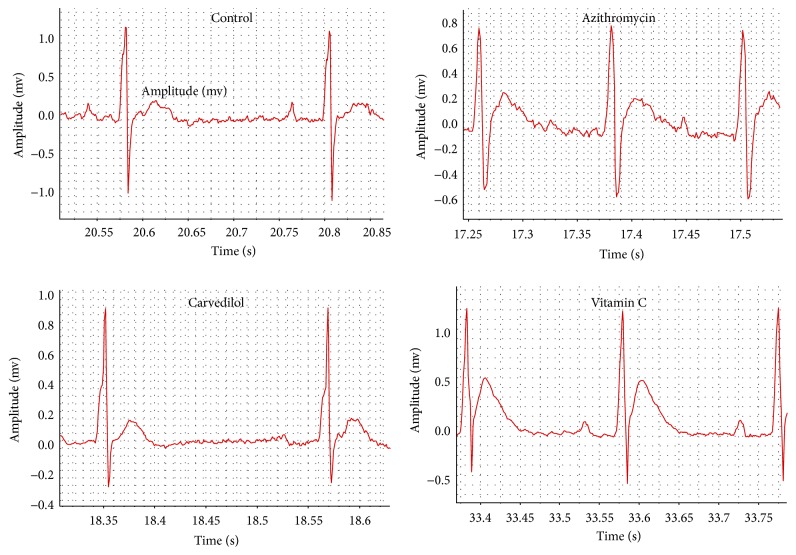
Effect of carvedilol and vitamin C on azithromycin-induced changes in the electrocardiographic (ECG) pattern measured in rats.

Pretreatment of azithromycin-treated rats with both carvedilol (10 mg/kg) and vitamin C (20 mg/kg) protected the ECG patterns from most of the azithromycin-induced effects (Figure 2). Pretreatment of azithromycin-treated rats with both carvedilol and vitamin C significantly decreased the heart rate (*p* = 0.000  and  0.031, resp.), QT interval (*p* = 0.024  and  0.008, resp.), and QTc (*p* = 0.002  and  0.014, resp.) compared to the azithromycin values (Table 1). On the other hand, pretreatment of azithromycin-treated rats with both carvedilol and vitamin C significantly increased the RR interval (*p* = 0.000  and  0.020) compared to the azithromycin value (Table 1).

Pretreatment of azithromycin-injected rats with carvedilol significantly decreased the Tpeak-Tend interval (*p* = 0.018) and T amplitude (*p* = 0.025) compared to the azithromycin values (Table 1).

Pretreatment of azithromycin-treated rats with vitamin C significantly decreased the P duration (*p* = 0.017) compared to the azithromycin value (Table 1).

Pretreatment of azithromycin-treated rats with both carvedilol and vitamin C could not decrease the ST elevation induced by the azithromycin treatment (Table 1).

### 3.2. Effects of Carvedilol and Vitamin C on Plasma LDH and CPK Enzyme Activities Measured in Azithromycin-Treated Rats (Table 2)

Treatments of rats with azithromycin significantly increased both plasma LDH and CPK enzyme activities (130% and 263%, resp.) compared to the control values (*p* = 0.000).

Pretreatment of azithromycin-treated rats with both carvedilol (10 mg/kg) and vitamin C (20 mg/kg) significantly decreased plasma LDH enzyme activity (63% and 53%, resp.) compared to the azithromycin value (*p* = 0.001).

Pretreatment of azithromycin-treated rats with vitamin C significantly decreased plasma CPK enzyme activity (57%) compared to the azithromycin value (*p* = 0.001).

On the other hand, pretreatment of azithromycin-treated rats with carvedilol did not significantly reduce the LDH enzyme activity (12%) compared to the azithromycin value (*p* = 0.494).

### 3.3. Effects of Carvedilol and Vitamin C on Myocardial Muscle MDA, GSH, and NO Contents Measured in Azithromycin-Treated Rats

**Figure 3:**
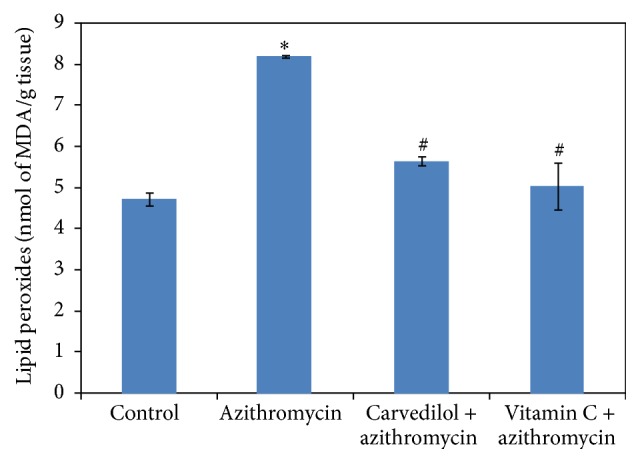
Effect of carvedilol and vitamin C on myocardial muscle lipid peroxide (MDA) content (nmol/g tissue) measured in azithromycin-treated rats. Data are presented as mean ± SDM (*n* = 6). ^*∗*^ Significant versus control (*p* ≤ 0.05). ^#^ Significant versus azithromycin (*p* ≤ 0.05).

**Figure 4:**
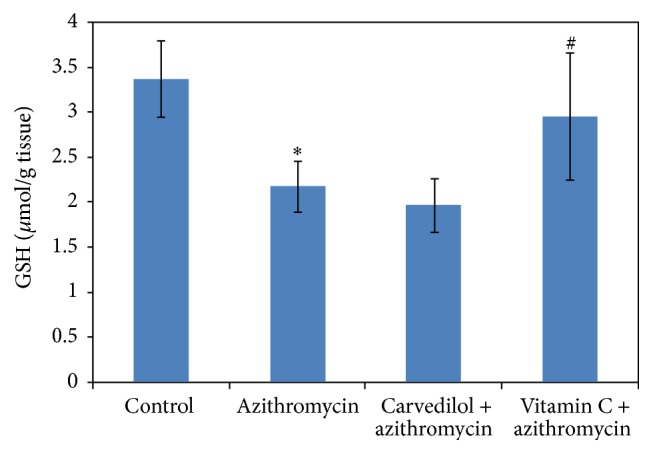
Effect of carvedilol and vitamin C on myocardial muscle GSH content (*μ*mol/g tissue) measured in azithromycin-treated rats. Data are presented as mean ± SDM (*n* = 6). ^*∗*^ Significant versus control (*p* ≤ 0.05). ^#^ Significant versus azithromycin (*p* ≤ 0.05).

**Figure 5:**
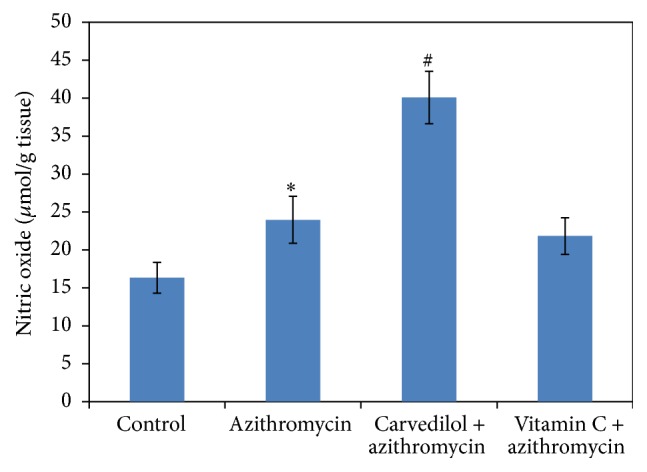
Effect of carvedilol and vitamin C on myocardial muscle nitric oxide (NO) content (*μ*mol/g tissue) measured in azithromycin-treated rats. Data are presented as mean ± SDM (*n* = 6). ^*∗*^ Significant versus control (*p* ≤ 0.05). ^#^ Significant versus azithromycin (*p* ≤ 0.05).

Treatments of rats with azithromycin significantly increased both MDA and NO contents (74% and 47%, resp.) compared to the control values (*p* = 0.000) (Figures 3 and 5). On the contrary, treatments of rats with azithromycin significantly decreased the GSH content (36%) compared to the control value (*p* = 0.000) (Figure 4).

Pretreatment of azithromycin-treated rats with both carvedilol and vitamin C significantly decreased the MDA content (31% and 39%, resp.) compared to the azithromycin values (*p* = 0.000) (Figure 3).

Pretreatment of azithromycin-treated rats with carvedilol did not significantly affect GSH content (10%) compared to the azithromycin value (*p* = 0.184). On the other hand, pretreatment of azithromycin-treated rats with vitamin C significantly increased GSH content (36%) compared to the azithromycin value (*p* = 0.030) (Figure 4).

Pretreatment of azithromycin-treated rats with carvedilol significantly increased the NO content (67%) compared to the azithromycin value (*p* = 0.000). On the other hand, pretreatment of azithromycin-treated rats with vitamin C did not significantly affect the NO content (8%) compared to the azithromycin value (*p* = 0.213) (Figure 5).

### 3.4. Effects of Carvedilol and Vitamin C on Myocardial Muscle GPx, SOD, and CAT Enzyme Activities Measured in Azithromycin-Treated Rats (Table 3)

There was no change in CAT enzyme activity in all treatment regimens. Treatments of rats with azithromycin significantly decreased GPx and SOD activities (49% and 54%, resp.) compared to the control values (*p* = 0.007 and 0.018, resp.).

Pretreatments of azithromycin-treated rats with carvedilol significantly increased GPx activity (165%) compared to the control value (*p* = 0.005). On the other hand, pretreatment of azithromycin-treated rats with carvedilol caused a nonsignificant decrease in SOD activity (22%) compared to the azithromycin value (*p* = 0.490). In addition, pretreatment of azithromycin-treated rats with vitamin C significantly increased GPx and SOD activity (85% and 89%, resp.) compared to the azithromycin values (*p* = 0.009 and 0.015, resp.).

### 3.5. Effects of Carvedilol and Vitamin C on Plasma IL-1*β* and TNF-*α* Measured in Azithromycin-Treated Rats (Figures 6 and 7)

Treatments of rats with azithromycin significantly increased both IL-1*β* and TNF-*α* (4-fold and 2.5-fold, resp.) compared to the control values (*p* = 0.000). Pretreatments of azithromycin-treated rats with both carvedilol and vitamin C significantly decreased IL-1*β* (30% and 29%, resp.) (*p* = 0.001 and 0.003, resp.) and TNF-*α* (57% and 41%, resp.) (*p* = 0.001) compared to the control values.

### 3.6. Effects of Carvedilol and Vitamin C on the Myocardial Muscle Histopathological Changes Detected by H&E Staining in Azithromycin-Treated Rats

**Figure 8:**
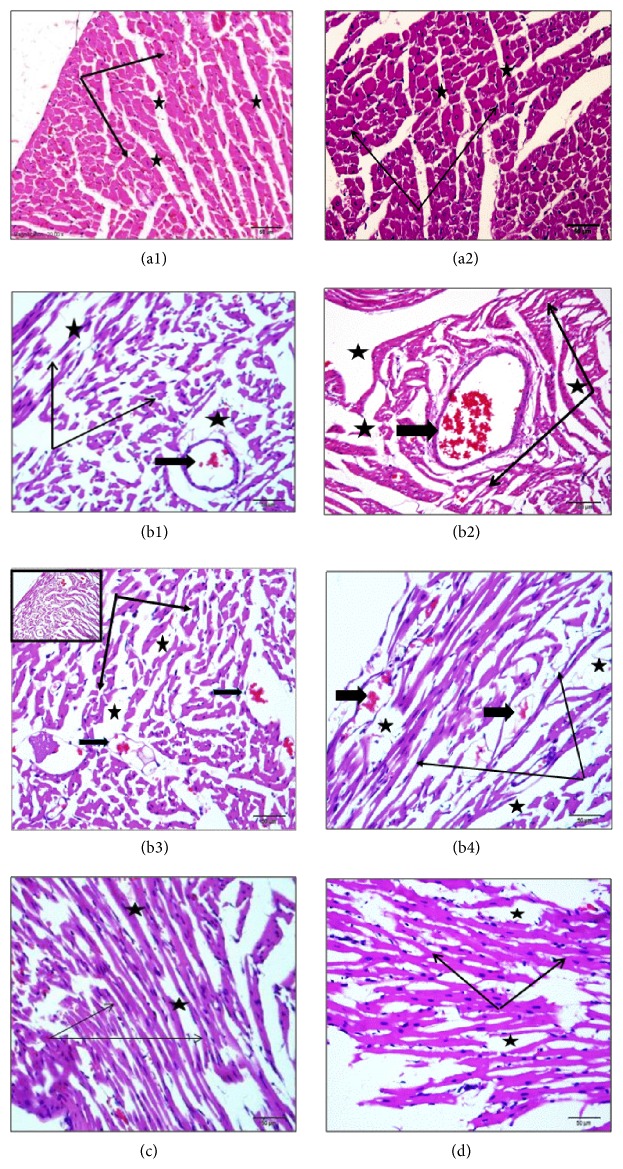
Effect of carvedilol and vitamin C on the myocardial muscle histopathological changes detected by H&E staining in azithromycin-treated rats. Light micrograph of rat heart ((a1) and (a2)). Control showing intact muscle fibers arranged as bundles (thin arrows) with normal connective tissue spaces (stars). ((b1)–(b4)) Azithromycin-treated rats showing marked atrophy of cardiac muscle fibers (thin arrows) with increased tissue spaces (stars), and coronary arteries are also dilated and congested and even ruptured (thick arrows) within the atrophy of their muscular walls. (c) Carvedilol + azithromycin-treated rats showing absence of capillary congestion and hemorrhage with marked preservation of cardiomyocyte morphology and tissue space (arrows and stars). (d) Vitamin C + azithromycin-treated rats showing neither congestion nor hemorrhage but still a moderate presence of cardiac muscle atrophy (sections are H&E stained ×20).

Treatment of rats with azithromycin resulted in marked atrophy of cardiac muscle fibers with increased tissue spaces and dilated, congested, and even ruptured coronary arteries with atrophied muscular walls (Figures 8(b1)–8(b4)).

Pretreatment of azithromycin-treated rats with carvedilol resulted in the absence of capillary congestion and hemorrhage, with marked preservation of cardiomyocyte morphology and tissue space (Figure 8(c)). In addition, pretreatment of azithromycin-treated rats with vitamin C resulted in the absence of capillary congestion and hemorrhage, but moderate cardiac muscle atrophy was still noticed (Figure 8(d)).

### 3.7. Effects of Carvedilol and Vitamin C on the Myocardial Muscle Caspase-3 Expression in Azithromycin-Treated Rats

**Figure 9:**
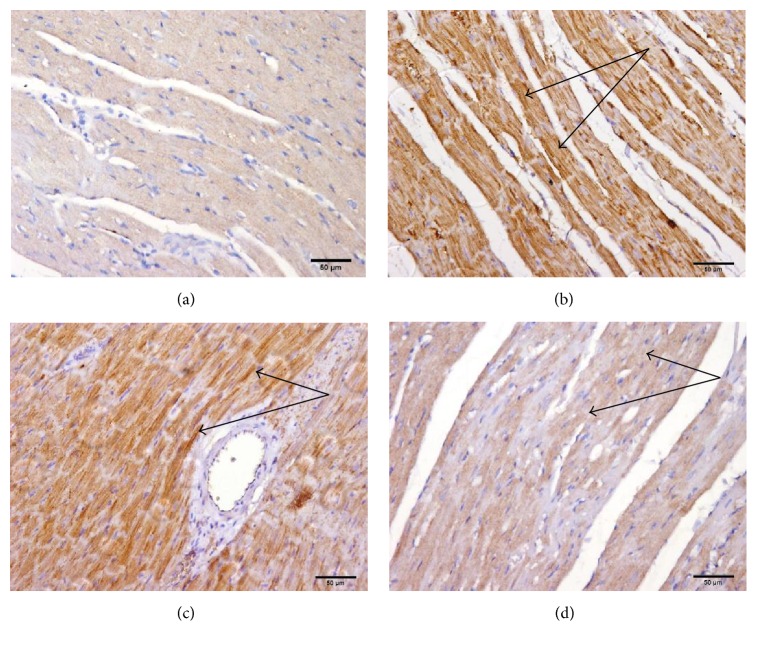
Effect of carvedilol and vitamin C on the myocardial muscle caspase-3 expression determined in azithromycin-injected rats. (a) Control showing negative (−) staining for caspase-3; (b) azithromycin-treated rats showing marked (+++) caspase-3 positivity; (c) carvedilol + azithromycin-treated rats showing moderate (++) caspase-3 positivity; (d) vitamin C + azithromycin-treated rats showing mild (±) caspase-3 positivity (sections are immunohistochemically stained ×20).

Treatment of rats with azithromycin resulted in marked caspase-3 expression (Figure 9(b)). Pretreatment of azithromycin-treated rats with carvedilol resulted in moderate caspase-3 expression (Figure 9(c)). Pretreatment of azithromycin-treated rats with vitamin C resulted in mild caspase-3 expression (Figure 9(d)).

### 3.8. Effects of Carvedilol and Vitamin C on the Myocardial Muscle NF-*κ*B Expression in Azithromycin-Treated Rats

**Figure 10:**
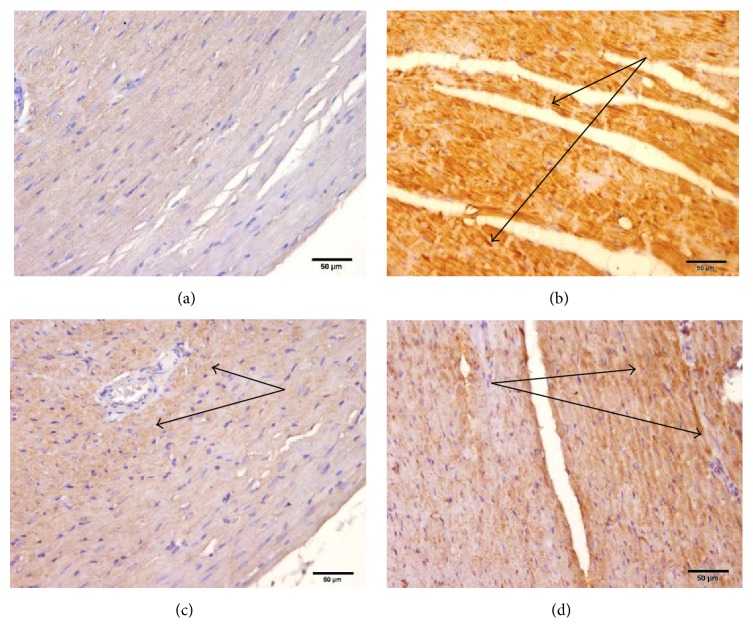
Effect of carvedilol and vitamin C on the myocardial muscle NF-*κ*B expression in azithromycin-injected rats. (a) Control showing negative (−) staining for NF-*κ*B; (b) azithromycin-treated rats showing marked (+++) NF-*κ*B positivity; (c) carvedilol + azithromycin-treated rats showing negative (−) NF-*κ*B positivity; (d) vitamin C + azithromycin-treated rats showing moderate (++) NF-*κ*B positivity (sections are immunohistochemically stained ×20).

Treatment of rats with azithromycin resulted in marked NF-*κ*B expression (Figure 10(b)). Pretreatment of azithromycin-treated rats with carvedilol resulted in no NF-*κ*B expression (Figure 10(c)). Pretreatment of azithromycin-treated rats with vitamin C resulted in moderate NF-*κ*B expression (Figure 10(d)).

## 4. Discussion

This study examined the impact of a 5-day azithromycin (10 mg/kg) course on the ECG components, cardiac enzymes, and histology of the rat heart compared to the control rat heart. We found that administration of azithromycin (10 mg/kg) for 5 days in rats has been associated with increased heart rate, P duration, QT interval, QTc, Tpeak-Tend interval, ST height, and T amplitude. No cases of torsades de pointes were noticed in this study. Azithromycin also increased plasma LDH and CPK enzyme activity. In addition, it induced major histopathological changes in the heart and blood vessels. Recently, most of our results were similarly reported by Atli et al. [16]. They documented that administration of both 15 and 30 mg/kg azithromycin for 14 days induced cardiotoxicity in rats.

Prolonged QT and QTc intervals are related to cardiac vagal dysfunction, representing cardiac toxic potential as increased risk of ventricular arrhythmia, cardiac dysfunction, and sudden cardiac collapse [35, 36]. Azithromycin-induced ventricular arrhythmias could be linked to Tpeak-Tend interval prolongation [37–40]. This study showed that azithromycin increased Tpeak-Tend intervals, which may account for increased mortality in patients with QT prolongation.

ST-segment elevation occurred with azithromycin administration, revealing that damaged cardiac muscle undergoes pathological changes and inflammation. The ECG changes induced by azithromycin could be due to the marked atrophy of cardiac muscle fibers and increased tissue spaces. These pathological changes are likely due to oxidant stress-induced repolarization changes, which may be an indicator of more severe damage, thus increasing cardiac susceptibility to cardiotoxicants [41].

Azithromycin increases T wave amplitude, which is one of the earliest ECG changes following coronary artery occlusion and ischemia. In line with the increased T wave amplitude, this study indicates atrophy within coronary artery muscular walls [42].

Our results show that azithromycin-induced cardiac complications are accompanied by induction of cardiac muscle oxidative stress, as measured by the increase in MDA, NO, and NF-*κ*B expression and the decrease in GSH, GPx, and SOD. Azithromycin also increases inflammatory response as it increases IL-1*β* and TNF-*α*. Moreover, azithromycin increases caspase-3 immunoreactivity. This indicates that azithromycin-induced cardiac adverse events might be due to disturbances in the antioxidant/oxidant balance, apoptotic cell-death, and/or immunomodulation.

Our results show that azithromycin increases myocardial GSH content. Similar results are reported with adriamycin injection in rats [43, 44]. This could be a defense mechanism against azithromycin-induced toxicity [45]. The decreased activity of both GPx and SOD in the azithromycin-treated rats might be due to increased generation of ROS [16, 41].

TNF-*α* was known to attract leukocytes to the inflammatory sites, enhancing the generation of reactive species [46]. Moreover, TNF-*α* seemed to be responsible for regulating the production of some mediators that stimulate inflammatory reactions, such as NF-*κ*B and inducible cyclooxygenase enzyme (COX-2) [47].

NO was found to play a controversial role in the heart. Rassaf et al. [48] reported that NO is an important vasodilator produced by coronary endothelial cells, which exert a cardioprotective effect. It serves as an oxygen free-radical scavenger, hence minimizing the deleterious effects of oxidative stress. However, Ishiyama et al. [49] reported that increased NO production by inducible NO synthase (iNOS) might be contributing to negative inotropic effects and the progression of myocardial damage [50]. In addition, many previous studies showed that increased TNF-*α* production induces iNOS and therefore NO formation in the myocytes. Azithromycin might increase myocardial NO content via TNF-*α*-mediated pathways.

The increased free-radical formation and the attenuation of antioxidant defenses by azithromycin could lead to oxidative damage of cellular lipids, proteins, and DNA [50]. This could explain the observed increase in the expression of NF-*κ*B, the nuclear factor that contributes to inflammatory response and cell apoptosis. Moreover, the results show increased expression of caspase-3 in the cardiac tissues of azithromycin-treated rats. Caspase-3 is an important apoptotic marker, and this finding indicates that azithromycin-induced cardiac effects could lead to the apoptosis of cardiac cells [51].

Concomitant administration of carvedilol protected the rats against most of the ECG pattern alterations induced by azithromycin. Our results were in line with those of Yildirir et al. [52]. The present study clearly demonstrates that carvedilol prevents azithromycin-induced QT prolongation [53]. The adrenergic blocking action might account for the QT dispersion reduction caused by carvedilol [54].

The carvedilol group showed no alteration in the integrity of the myocardium reflected by normal ECG segments (normal RR interval) [55]. Azithromycin showed attenuation of the RR interval (which indicates myocardial edema and loss of membrane integrity) and raised heart rates [56].

However, this study shows that carvedilol decreases neither P duration, ST height, nor CPK levels, and hence the ischemic risks of azithromycin might still be present. From our results, we concluded that the positive effects of carvedilol might be due to inhibition of free-radical formation, inflammatory mediators (TNF-*α* and IL-1*β*), and apoptotic cell-death (caspase-3) [52, 57].

In this study, carvedilol stimulated nitric oxide synthesis in rats' cardiac myocytes [58]. Takano et al. [59] reported a cardioprotective effect of iNOS in an experimental model of myocardial infarction. Therefore, the enhanced NO production by carvedilol might act as a cardioprotective effect in myocardial ischemia induced by azithromycin [60].

Pretreatment of azithromycin-treated rats with vitamin C protected the rats against most of the azithromycin-induced ECG pattern alterations. Vitamin C decreased neither Tpeak-Tend intervals nor ST height. In addition to the improvement of serum cardiac enzymes, vitamin C also ameliorated the altered oxidative stress biomarkers, suggesting that the cardioprotective effect of vitamin C is due at least in part to its antioxidant and free-radical scavenging activity. Vitamin C also decreased the elevated IL-1*β* and TNF-*α* and attenuated the caspase-3 expression, suggesting anti-inflammatory and antiapoptotic cardioprotective mechanisms against azithromycin-induced cardiotoxicity. Our results matched those of Abdel-Daim et al. [61] and Yavuz et al. [62].

This study concludes that azithromycin may produce oxidative stress via the generation of highly toxic oxygen and nitrogen free-radicals and the inhibition of several antioxidant enzymes. Azithromycin may also increase inflammation and apoptosis. Hence, it causes ischemic necrosis, leading to alterations in ECG segments, thereby altering several physiological and functional changes in the heart; this resembles human myocardial infarction, which causes sudden death in humans. Both carvedilol and vitamin C administration showed their cardioprotective effects through significant attenuation of azithromycin-induced ECG alterations, which might be attributed to their antioxidant, anti-inflammatory, and antiapoptotic effects; thus, they maintained the integrity and permeability of the cellular membranes.

## 5. Recommendation

Further experimental studies are recommended to explore the precise mechanism(s) of azithromycin-induced cardiac complications and sudden death in humans.

## Figures and Tables

**Figure 6:**
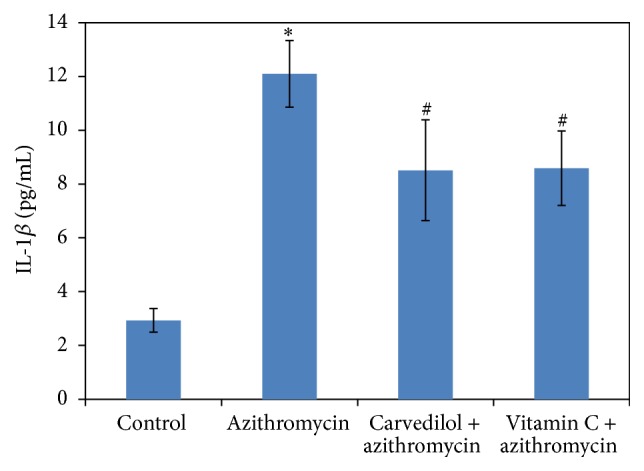
Effect of carvedilol and vitamin C on plasma IL-1*β* concentrations (pg/mL) measured in azithromycin-treated rats. Data are presented as mean ± SDM (*n* = 6). ^*∗*^ Significant versus control (*p* ≤ 0.05). ^#^ Significant versus azithromycin (*p* ≤ 0.05).

**Figure 7:**
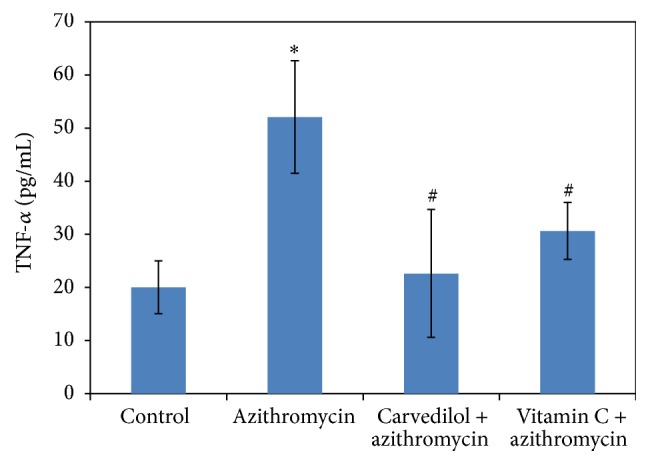
Effect of carvedilol and vitamin C on plasma TNF-*α* concentrations (pg/mL) measured in azithromycin-treated rats. Data are presented as mean ± SDM (*n* = 6). ^*∗*^ Significant versus control (*p* ≤ 0.05). ^#^ Significant versus azithromycin (*p* ≤ 0.05).

**Table 1:** Effect of carvedilol and vitamin C on the electrocardiographic (ECG) parameters measured in azithromycin-treated rats.

ECG parameters	Control	Azithromycin	Carvedilol + azithromycin	Vitamin C + azithromycin
Heart rate (BPM)	310 ± 28	447 ± 32^a^	330 ± 46^b^	372 ± 67^b^
P duration (s)	0.012 ± 0.003	0.015 ± 0.001^a^	0.017 ± 0.005	0.012 ± 0.002^b^
RR interval (s)	0.18 ± 0.03	0.13 ± 0.01^a^	0.19 ± 0.02^b^	0.17 ± 0.03^b^
PR interval (s)	0.04 ± 0.007	0.05 ± 0.008	0.05 ± 0.002	0.05 ± 0.003
QRS interval (s)	0.016 ± 0.002	0.016 ± 0.004	0.016 ± 0.001	0.016 ± 0.004
QT interval (s)	0.04 ± 0.012	0.07 ± 0.01^a^	0.06 ± 0.012^b^	0.06 ± 0.005^b^
QTc (s)	0.115 ± 0.02	0.194 ± 0.03^a^	0.128 ± 0.02^b^	0.146 ± 0.02^b^
Tpeak-Tend interval (s)	0.019 ± 0.007	0.041 ± 0.016^a^	0.022 ± 0.013^b^	0.029 ± 0.010
ST height (mV)	−0.21 ± 0.10	0.022 ± 0.19^a^	−0.013 ± 0.08	0.04 ± 0.15
T amplitude (mV)	0.16 ± 0.09	0.28 ± 0.09^a^	0.14 ± 0.09^b^	0.29 ± 0.18
Q amplitude (mV)	−0.02 ± −0.04	−0.002 ± −0.01	−0.002 ± −0.01	−0.008 ± −0.02
S amplitude (mV)	−0.52 ± −0.33	−0.59 ± −0.19	−0.27 ± −0.13^b^	−0.42 ± −0.22
P amplitude (mV)	0.08 ± −0.05	0.06 ± −0.01	0.06 ± −0.013	0.09 ± −0.06
R amplitude (mV)	1.03 ± 0.42	1.09 ± 0.38	0.89 ± 0.08	0.99 ± 0.47

Data are presented as mean ± SDM (*n* = 6).

^a^Significant versus control (*p* ≤ 0.05).

^b^Significant versus azithromycin (*p* ≤ 0.05).

**Table 2:** Effect of carvedilol and vitamin C on plasma lactate dehydrogenase (LDH) and creatine phosphokinase (CPK) enzyme activity measured in azithromycin-treated rats.

Groups	LDH (U/L)	CPK (U/L)
Control	181 ± 77	62 ± 17
Azithromycin	417 ± 84^a^	225 ± 61^a^
Carvedilol + azithromycin	156 ± 109^b^	197 ± 75
Vitamin C + azithromycin	195 ± 125^b^	97 ± 32^b^

Data are presented as mean ± SDM (*n* = 6).

^a^Significant versus control (*p* ≤ 0.05).

^b^Significant versus azithromycin (*p* ≤ 0.05).

**Table 3:** Effect of carvedilol and vitamin C on cardiac muscle glutathione peroxidase (GPx), superoxide dismutase (SOD), and catalase (CAT) enzyme activity measured in azithromycin-treated rats.

Groups	GPx (U/g tissue)	SOD (U/g tissue)	CAT (U/g tissue)
Control	278 ± 73	881 ± 339	0.26 ± 0.09
Azithromycin	142 ± 65^a^	409 ± 229^a^	0.26 ± 0.07
Carvedilol + azithromycin	376 ± 147^b^	319 ± 206	0.32 ± 0.19
Vitamin C + azithromycin	262 ± 63^b^	774 ± 202^b^	0.20 ± 0.13

Data are presented as mean ± SDM (*n* = 6).

^a^Significant versus control (*p* ≤ 0.05).

^b^Significant versus azithromycin (*p* ≤ 0.05).
